# Identification of Key Genes and Pathways Associated with Age-Related Macular Degeneration

**DOI:** 10.1155/2020/2714746

**Published:** 2020-08-21

**Authors:** Junyu Zhang, Yu Zhou

**Affiliations:** ^1^Sichuan Provincial Key Laboratory for Human Disease Gene Study, Sichuan Provincial People's Hospital, School of Medicine, University of Electronic Science and Technology of China, Chengdu 611731, China; ^2^International Peace Maternity and Child Health Hospital, School of Medicine, Shanghai Jiao Tong University, Shanghai, China; ^3^Department of Pathology, Brigham and Women's Hospital, Harvard Medical School, Boston, Massachusetts, USA; ^4^Department of Laboratory Medicine, Sichuan Academy of Medical Sciences & Sichuan Provincial People's Hospital, Chengdu 610072, China; ^5^Department of Cancer Biology, Dana-Farber Cancer Institute, Boston, Massachusetts 02215, USA; ^6^Department of Genetics, Harvard Medical School, Boston, Massachusetts 02115, USA

## Abstract

Age-related macular degeneration (AMD) is the leading cause of severe, permanent vision loss among the elderly in the developed world. The cellular and molecular pathogenesis of initiation and development of AMD remain poorly delineated. The limited resources of the human AMD RPE/choroid tissues impeded the extensive study of the disease. To better understand the molecular and pathway changes in human AMD RPE/choroid tissues, we searched the literature and found three independent studies using high-throughput technology to analyze gene expression in 54 human AMD RPE/choroid tissues and 46 age-matched healthy controls. We downloaded these data, pooled them together, and reanalyzed the difference between molecular and pathways by the Ingenuity Pathway Analysis (IPA) database. Totally, 353 differentially expressed genes (DEGs) were identified, among which 181 genes were downregulated and 172 genes were upregulated in RPE/choroid of AMD patients. Furthermore, several significantly enriched biological processes, including cancer, organismal injury and abnormalities, and ophthalmic disease, were identified associated with these DEGs. By analysis of canonical pathway, the phototransduction pathway and atherosclerosis signaling were the top two significant canonical pathways altered in RPE/choroid tissues in human AMD. As expected, several ophthalmic disease-related molecules, including RHO, PDE6A, 3′,5′-cyclic-GMP phosphodiesterase, and G protein alpha, were in the central nodes of disease network. The bioinformatics technology combined with the existing high-throughput data was applied to evaluate the underlying key genes and pathways in human AMD tissues, which may predict downstream and upstream biological processes and identify potential therapeutic intervention targets in human AMD.

## 1. Introduction

Age-related macular degeneration (AMD), also called macular degeneration, AMD or ARMD, is a leading cause of severe vision loss in the elder individuals. The incidence of the disease relates with aging and exponentially increases every ten years after age 50 years [1]. By 2040, nearly 288 million people are expected to be affected [2]. The progress of AMD is gradually from early and intermediate stage through to late stage. Two main types of AMD, geographic atrophy (GA, “dry” AMD) and/or choroidal neovascularization (CNV, “wet” AMD), are manifested during the development of AMD. While wet AMD can be treated, unfortunately, there is no treatment for dry AMD forms [3]. AMD is believed to have a multifactorial etiology, which can be caused by a complex interplay of genetic, metabolic, environmental, and functional factors [4]. In spite of advances in last decade's diagnosis, treatment, and understanding of AMD, the disease prevalence and economic cost will steadily increase in the next decades.

Retinal pigment epithelium (RPE), a specialized epithelium lying between the choriocapillaris and the neural retina, is initially affected in AMD, resulting in a subsequent loss of photoreceptor cells over time [3]. RPE performs a variety of optical, physical, metabolic, and transport functions that are essential for neural retina homeostasis [5]. Drusen deposits and accumulates between the RPE and Bruch's membrane caused by impaired RPE function in dry AMD [6]. Choroidal neovascularization is involved in wet AMD, followed by the formation of a disciform scar [7]. Compared with normal RPE, AMD RPE is more susceptible to oxidative stress, which shows reduced mitochondrial activity and higher levels of reactive oxygen species (ROS) under stress conditions [8]. Considering the critical role of the RPE, gene mutations found in this epithelium or gene alterations in their expression secondary to environmental changes would probably contribute to the pathogenic mechanism of AMD. Although the profiles of RPE gene expression in mouse and fatal human tissues have been established [9, 10], large-scale functional genomics data sets in human tissues between AMD patients and healthy controls are still limited.

Elucidation of molecular and pathways in RPE/choroid leading to AMD development and progression in patients is difficult to achieve, as the study involves decade observation. Sometimes the samples can only be acquired after the donor deceased. These studies are also hampered by the need for the patients under surveillance to obtain repeated biopsies. High-throughput gene expression analysis technology, including microarray and RNA-seq, can give us a broad and comprehensive view to the molecular changes in some precious biopsies [11], such as human RPE/choroid, with high efficiency. Consequently, it is advantageous and worthwhile to pool the existing high-throughput gene expression data in RPE/choroid of human AMD and deeply evaluate the differential gene expression and their association to AMD.

In order to generate a comprehensive profile of key genes and pathways altered in human RPE/choroid of AMD, we pooled the three published GEO data (GSE29801 [12], GSE50195 [13], and GSE99248 [14]) and used the bioinformatictools to disclose DEGs and pathways and reveal factors, especially central networks, downstream effects, and upstream regulators in the development and procession of AMD. The key genes and pathways revealed in human RPE/choroid could be potential targets in AMD prevention and therapy.

## 2. Materials and Methods

### 2.1. Sources of Data

The transcriptome profile datasets involving gene expression in RPE/choroid tissues of human AMD tissues were obtained from NCBI GEO databases (http://www.ncbi.nlm.nih.gov/geo/). Based on the principle that all the data were from human RPE/choroid tissues of AMD and age-matched healthy controls, three GEO data (GSE50195, GSE99248, and GSE29801) were included in the present study. The microarray analysis of GSE50195 was evaluated by Affymetrix GeneChip Human Exon ST 1.0 arrays. The RNA-seq data of GSE99248 were performed on Illumina HiSeq2000 from prepared RNA libraries. The transcriptome profiling of GSE29801 was carried out using the following system 4 × 44 K in situ oligonucleotide array platform Agilent Whole Human Genome.

### 2.2. Data Processing Pipeline and Differential Gene Expression

The GSE50195 and GSE99248 were reanalyzed using the R software (version 3.4.0; https://www.r-project.org/) and Bioconductor packages (http://www.bioconductor.org/). The quality of samples and data has been well controlled in the original study. For the original array data, the context correction and normalization were performed using the robust multiarray average process. RNA-seq data were downloaded from the GEO database, which have been all normalized, and the reference-based assembly for gene expression calculation has been carried out. Afterward, in the R affy package, they were subsequently converted into expressive measures. DEGs were subsequently identified using the Limma package. The cutoff values for DEG screening using the Benjamini and Hochberg procedure were considered to be *p* ≤ 0.01 and absolute Log_2_Ratio ≥1. The tables of the original differentially expressed gene of GSE29801 were downloaded. The cutoff values for DEG screening were set as *p* ≤ 0.01 and absolute Log_2_Ratio ≥1. The quality of samples and data has been well controlled in the original study. Finally, using the Bioconductor package Venn diagram, the differentially expressed genes among GSE50195, GSE99248, and GSE29801 in RPE/choroid between AMD patients and controls were identified. The cutoff values were considered to be *p* ≤ 0.05 and absolute Log_2_Ratio ≥1.

### 2.3. Functional Analysis, Canonical Pathway Analysis, and Generation of Networks

Ingenuity pathway analysis (IPA) (Qiagen) has been used to identify top biological functions, as well as canonical pathways associated with DEGs from GSE29801, GSE50195, and GSE99248. Fisher's exact test was carried out to calculate the probability if any biological function or canonical pathway could be explained by chance alone. Algorithmically, molecular interaction networks are created based on the connectivity of the molecules. Network scores were determined using Fisher's exact test and corresponded to −log 10 (*p* value).

### 2.4. Downstream Effects Analysis and Upstream Regulator Analysis

Analysis of downstream effects was used to understand downstream biological processes and at the same time to predict their increased or decreased activation status based on changes in the datasets from the observed gene expression. To infer the status of activation (“increased” or “decreased”) of the biological processes involved, a z-score was measured. A *p* value ≤ 0.05 overlap enrichment of network-regulated genes has been defined in the dataset. Probable regulating molecules based on a statistically significant up- and downregulation pattern match was identified by using a z-score ≤−2.0 or ≥2.0, including to predict either the activated or inhibited status of a putative regulator.

## 3. Results

### 3.1. Differentially Expressed Genes in RPE/Choroid between AMD Patients and Healthy Controls

**Figure 1:**
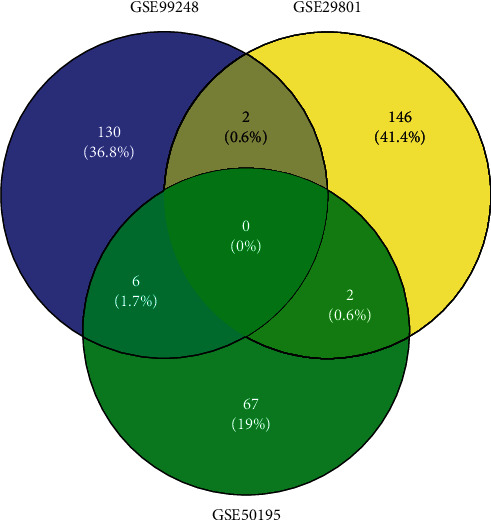
The commonly and differentially expressed genes in GSE29801, GSE50195, and GSE99248 were identified using Bioconductor package Venn diagram. *p* value ≤ 0.01 and Log_2_Ratio change ≥ 1 were considered as the cutoff values.

The gene expressions of human RPE/choroid tissues from 54 AMD patients and 46 age-matched controls in three different independent studies were enrolled in our study (Table 1). The summary and details of clinical information of the normal control and AMD patients is shown in Supplementary Table 1 and Supplementary Table 2, respectively. In the three studies, altered gene expression in RPE/choroid tissue between AMD patients and controls were analyzed by various platforms, including transcriptome, microarray, and RNA-seq analysis. We reanalyzed these data using *p* < 0.01 and absolute Log_2_Ratio ≥1 as the cutoff. Duplicates were consolidated using the maximum absolute log ratio. Finally, 353 DEGs were identified, among which 172 were upregulated (*p* ≤ 0.01 and Log_2_Ratio ≥1) and 181 (*p* ≤ 0.01 and Log_2_Ratio ≤ −1) were downregulated (Supplementary Table 3).  Figure 1 shows the differentially and commonly expressed genes among GSE29801, GSE50195, and GSE99248. Unfortunately, we did not find any gene overlapped in all of the studieshareds, but there were 2 shared DEGs in GSE29801 and GSE50195 (ARL9 and EFCAB1), 2 shared DEGs in GSE29801 and GSE99248 (WIF1 and TF), and 6 shared DEGs in GSE50195 and GSE99248 (MAP2, SV2B, RP1, KCNB1, IMPG1, and PDC).

### 3.2. Functional Analysis for DEGs in RPE/Choroid between AMD Patients and Healthy Controls

Ingenuity pathway analysis (IPA) (Qiagen) was used to explore the biological functions enriched in the DEGs. Several significantly enriched biological processes associated with DEGs were identified by IPA (Table 2 and Supplementary Table 4). Cancer (*p* value: 2.41*E*−03 to 5.96*E*−16, 299 genes assigned), organismal injury and abnormalities (*p* value: 2.41*E*−03 to 5.96*E*−16, 301 genes assigned), and ophthalmic disease (*p* value: 2.26*E*−03 to 9.88*E*−16, 58 genes assigned) were identified as the top three significantly enriched terms in the perspective of diseases and disorders. Cell morphology (*p* value: 2.19*E*−03 to 1.01*E*−12, 56 genes assigned), cellular movement (*p* value: 2.19*E*−03 to 1.82*E*−10, 84 genes assigned), and cellular compromise (*p* value: 1.88*E*−03 to 6.25*E*−10, 27 genes assigned) were found as the top three significantly enriched terms in the perspective of molecular and cellular functions. Nervous system development and function (*p* value: 1.88*E*−03 to 7.04*E*−14, 88 genes assigned), embryonic development (*p* value: 2.06*E*−03 to 1.01*E*−12, 55 genes assigned), and organ development (*p* value: 1.86*E*−03 to 1.01*E*−12, 48 genes assigned) were shown as the top three enriched terms in the perspective of physiological system development and functions.

### 3.3. Analysis of Canonical Pathway

**Figure 2:**
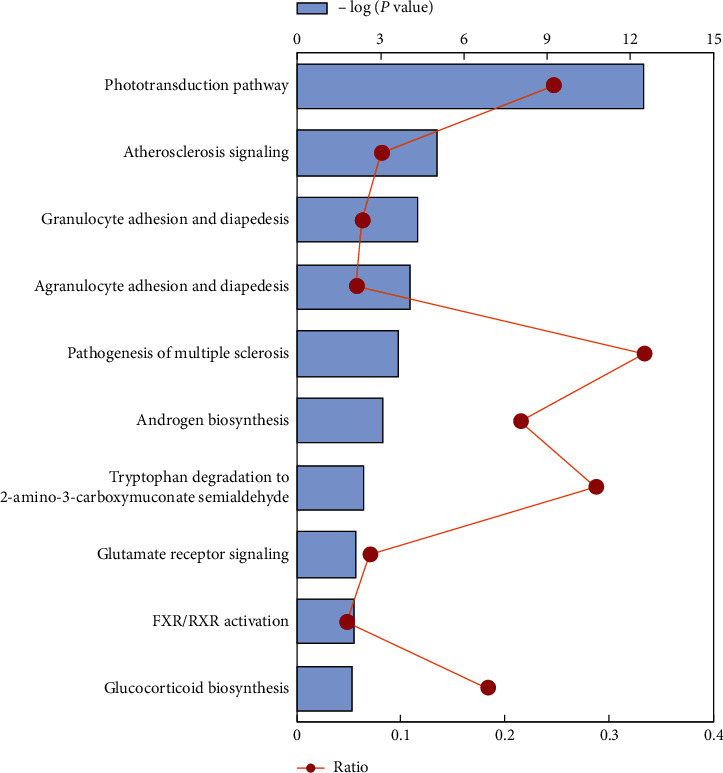
Ingenuity pathway analysis of the total significantly differentially expressed genes to identify the canonical pathways involved in RPE/choroid of AMD. The top ten significant pathways identified for the DEGs (blue bar). The orange curve shows the ratio between the number of DEGs and the total number of genes in each of these pathways (see the entire list of IPA pathways in Supplementary Table S5 online).

Canonical pathway analysis could inform the key metabolism and signaling pathways in which the DEGs may be involved. A total of 27 significant pathways were identified using the cutoff of *p*< 0.05.  Figure 2 shows the top 10 canonical pathways in RPE/choroid of AMD that significantly associated with DEGs. The phototransduction pathway (−log (*p* value) = 12.536) and atherosclerosis signaling (−log (*p* value) = 5.024) were the top two major pathways altered in RPE/choroid tissues in human AMD. The complete list of the IPA-identified pathways is given in Supplementary Table 5. The *p* values and Log_2_Ratio for each gene in top ten pathways are listed in Supplementary Table 6.

### 3.4. Interaction Network Analysis

**Figure 3:**
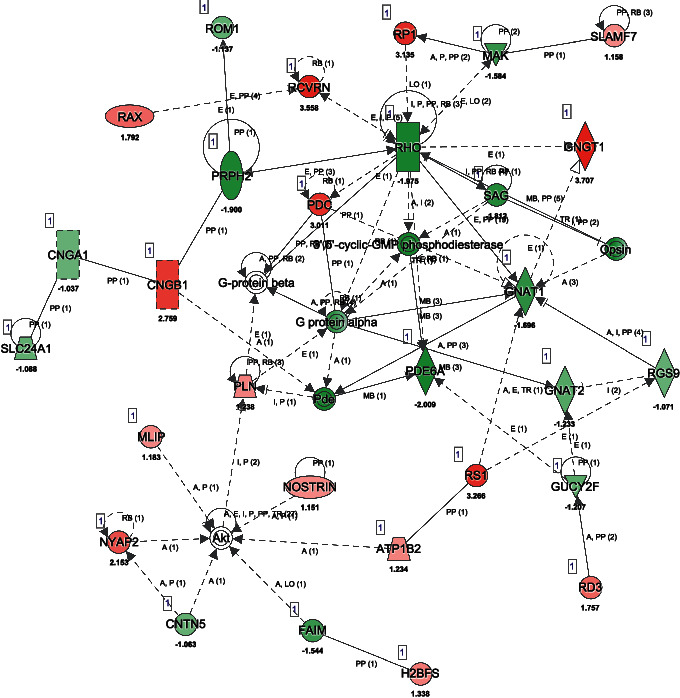
Molecular interaction networks: The most significant biological network of “hereditary disorder, neurological disease, organismal injury and abnormalities” was generated. Upregulated mRNAs are indicated in red, while downregulated mRNAs are in green. Solid lines represent the direct function, while the dotted lines represent the indirect function.

The molecular interaction networks were further generated using the connectivity of the identifiedDEGs and ranked by identified score. We suggested 23 networks, which were generated by the identified DEGs (Supplementary Table 7). The network of “hereditary disorder, neurological disease, and organismal injury and abnormalities” (score = 53) (Figure 3) was the most enriched network alerted in human RPE/choroid tissues of AMD. Notably, RHO, PDE6A, 3′,5′-cyclic-GMP phosphodiesterase, and G protein alpha were the central “nodes” with the greatest number of connections in the network. The *RHO* gene codes the protein rhodopsin, which is found in specialized light receptor cell rods and necessary for normal vision. Several *RHO* polymorphisms and haplotypes have been reported and confer remarkable sensitivity to AMD [15]. Heterotetrameric phosphodiesterase (PDE) 6 complex has an important function in the rod photoreceptor visual transduction cascade, which consists of *α*, *β*, and two *γ* subunits [16]. Mutation in *PDE6A* are one of the most common causes of arRP[17]. The 3′,5′-cyclic-GMP phosphodiesterase has other names in common use, including cyclic 3′,5′-GMP phosphodiesterase, cGMP phosphodiesterase, cGMP-PDE, etc. Recently, PDE inhibitors show promise in AMD treatment. G protein alpha, initiated by chemokine receptor binding to G-protein-coupled receptors (GPCRs), could activate second messenger molecules in cellular response. GPCRs are especially useful targets for the use of pharmacology systems, including the treatment of AMD [18].

### 3.5. Downstream Effects Analysis

**Figure 4:**
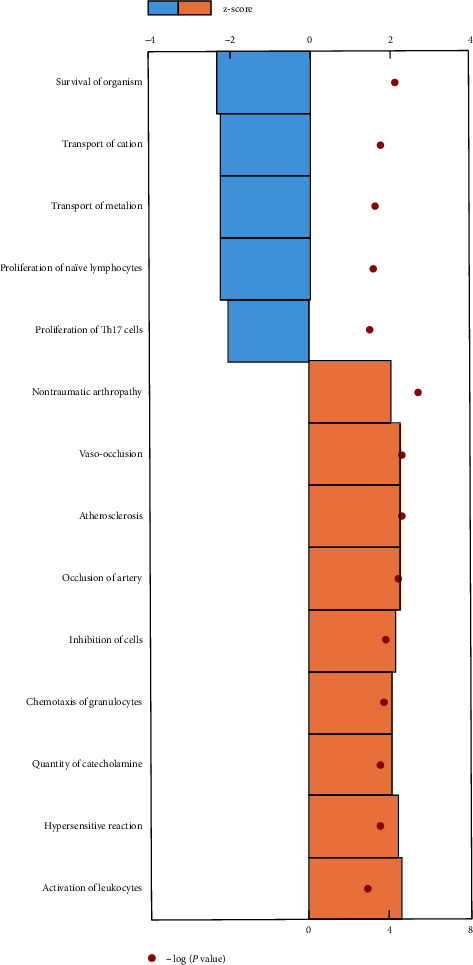
Downstream effects analysis. z-score and –log(*p* value) algorithm were used to identify biological functions that are expected to be increased/decreased in RPE/choroid between AMD patients and controls.

Biological processes and functions that are likely to be affected by the DEGs were analyzed by downstream effects analysis. Furthermore, downstream effects analysis also predicted the increased or decreased status of the biological processes. In the present study, both the z-score and *p* value were used to predict the downstream significant increased or decreased activation of 14 biological processes (Figure 4 and Supplementary Table 8). The increased concentration of cyclic AMP (z-score = 2.611; *P* = 1.65 × 10^−3^) and decreased survival of organism (z-score = −2.354; *P* = 3.64 × 10^−4^) were the most affected subcategories.

### 3.6. Upstream Regulator Analysis and Key Candidate Gene Identification

**Figure 5:**
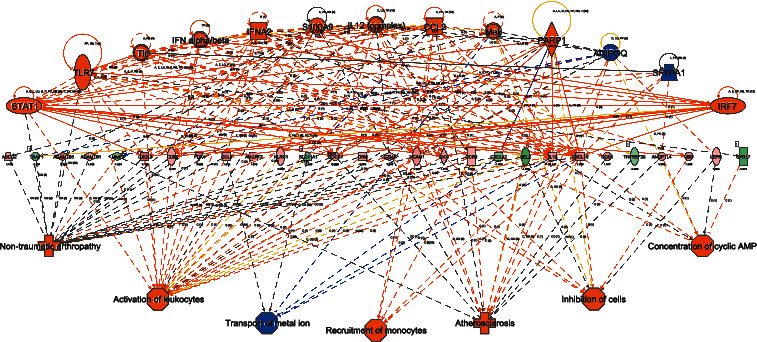
Upstream and regulator effect in RPE/choroid between AMD patients and controls. Genes in green were downregulated, and genes in red were upregulated. The deeper the color, the higher the degree of downregulation. Images in blue indicate that the decreased expression level of the upstream genes might suppress the expression of the downstream genes. Images in orange mean the decreased expression level of the upstream genes may lose their function as inhibitors of downstream effects. Full line in blue means the upstream genes can definitely regulate the expression of the downstream genes and effects. Lines in gray mean the upstream genes may have no effects on the regulation of downstream genes and effects.

The upstream transcriptional regulator includes microRNA, kinase, drug, compound, and transcription factor. In the current study, an activation z-score ≤−2.0 or ≥2.0 and an overlap *p* value ≤ 0.05 were used as the cutoff values. Excluding the chemicals and drugs, 32 upstream regulators were found, in which 7 upstream regulators were predicted to be inhibited while 27 upstream regulators were predicted to be activated. The molecular types of these upstream regulators were mainly cytokines, transcription regulators, complex, and enzyme. Notably, the immune and inflammation molecular, such as IFNa/*β*, IL12, CCL2, TLR7, and IFNA2,were identified in the upstream regulators, which highlight the importance of the immune response in the pathogenesis of AMD (Supplementary Table 9). To combine the upstream regulators, DEGs, and the downstream biological processes and functions, several regulator effect pathways were found in RPE/choroid between normal controls and AMD patients (Supplementary Table 10). The most significant regulator effect network is shown in Figure 5. The regulators in the upstream eventually contributeto the activation/inhibition of the biological processes, including activation of leukocytes, transport of metal ion, atherosclerosis, and the concentration of cyclin AMP.

## 4. Discussion

The retinal pigment epithelium (RPE) plays a key role in ocular development and maintenance of retinal homeostasis but has insufficient representation in large-scale gene expression and function datasets, especially in AMD human tissues. Although there were several gene expression and function studies in human AMD RPE/choroid tissues, neither of them were based on global gene expression throughput transcriptomic screening [19, 20], nor some of them were published with raw data [21]. Finally, we incorporated three published GEO data, and totally, 353 DEGs were identified after consolidating duplicates. However, no gene overlapped in all the studies, and even few genes overlapped with each other. This result suggests a genetic heterogeneous and phenotypic diversity characteristic of AMD [4]. Importantly, the samples of human AMD show significant interindependent variation in the expression of RPE transcript [22]. Thus, it is imperative to reveal connections of these DEGs, predict the upstream and downstream molecules/biological processes, and provide a panorama of those DEGs, which would help us have a comprehensive understanding of RPE pathogenesis in AMD and may suggest potential targetsfor AMD treatment.

Bioinformatic tools, such as IPA, can be used to identify gene modulations [23]. In this process, the biological pathways, networks, and functional effects related to the DEGs can be generated. In the current study, the functional analysis revealed 58 ophthalmic disease genes in the perspective of diseases and disorders. Interestingly, *IDO1* and *TDO2*, two key genes in tryptophan metabolism, recently were suggested to associate with autoimmune disease and tumor progression [24], prompt the immune response involved in the pathogenesis of RPE/choroid in AMD. In the perspective of molecular and cellular functions, “cell morphology” is the first top significantly enriched terms. Genes such as *RHO, CADM1, ELOVL4, GNAT1, GUCY2F, RP1*, and *SLC24A1* were inductive in this group. Most of them play an important role in development and metabolism in retinal rod and cone photoreceptors by mediating the protection of the retina from nutrient delivery, oxidative stress, ionic homeostasis, growth factors release, and ocular immune privilege establishment [13, 25–27]. Besides, nervous system development and function genes showed the most significant change in perspective of physiological system development and functions.

The RPE is essential for the photoreceptor's normal function and survival, although it is not the part of neural retina [28]. The RPE is the main site for 11-cis retinal regeneration in the visual cycle, among its other contributions to the photoreceptors [29]. Our data showed that the phototransduction pathway altered most in RPE/choroid in AMD. Absorbed photons are transformed into an electrical response in the process of phototransduction, which is highly preserved and based almost entirely on the function of opsins [30]. Several essential genes in the phototransduction pathway such as *CNGA1, CNGB1, GNAT1, GNAT2, GNGT1, GUCY2F, OPN1LW, PDC, PDE6A, RCVRN, RGS9, RHO,* and *SAG* were found in the DEGs. The second top altered pathway is atherosclerosis signaling. Although AMD risk variants in the genome-wide association analysis are also involved in atherosclerosis [31, 32], it is the first time to elucidate changed atherosclerosis signaling in RPE/choroid of AMD, which would combine the relationship between AMD and atherosclerosis from the aspect of gene expression.

“Hereditary disorder, neurological disease, and organismal injury and abnormalities” changed mostly in human RPE/choroid tissues of AMD, which was found by enriched network analysis. In these networks, RHO, PDE6A, 3′,5′-cyclic-GMP phosphodiesterase, and G protein alpha were in the central “nodes,” which prompt their roles as the target genes for AMD treatment. Interestingly, these molecules are being investigated as potentially potent pharmacological targets in AMD treatment. Rho-associated kinase (Rho-kinase/ROCK) inhibitors for glaucoma have already been clinically applied [33] and widely used for vitreoretinal disorders [34, 35]. In AMD treatment, after addition of Rho kinase inhibitors, retinal function and photoreceptor survival can be improved after subretinal cell delivery [36]. Rho kinase inhibitors also have a potential therapeutic benefit in neovascular AMD [37]. Phosphodiesterase (PDE) inhibitors, including Viagra (sildenafil; Pfizer [38]), Levitra (vardenafil; Bayer), and Cialis (tadalafil; Eli Lilly), continue to raise interest among eye specialists for the hypoperfusion-related ocular pathologic disease treatment, including diabetic retinopathy and AMD [39]. G protein alpha was released by the activation of GPCRs, which provided a strategy for AMD treatment from a system pharmacology aspect [18].

AMD lesions have shown through histopathological evaluation the involvement of inflammatory cells, including lymphocytes, macrophages, and mast cells [40], which is evidence that AMD is an immune-mediated disease. The RPE seems to be an accessory cell that might be important in the local immune response and crosstalk with vascular systems. In our present study, after analysis of downstream and upstream molecular as Figure 5 shown, the molecular type of these upstream regulators was mainly shown as cytokines and inflammation molecular, including IFNa/*β*, IL12, CCL2, TLR7, and IFNA2, which highlight the immune response in the initiation and progression of AMD. Indeed, AMD has been recognized as an immune-related disease like Alzheimer's and cardiovascular disease [41]. Furthermore, the downstream effects revealed an activated immune status in RPE/choroid tissues of AMD, such as “activation of leukocytes” and “recruitment of monocytes,” in which the cytokines or chemokines would be produced and positive feedback to the upstream molecular, thus cascade amplify the immune response and aggravate the RPE injury. Our data could help reveal the immune status change in RPE/choroid tissues of AMD not only in the upstream but also in the downstream, which would orchestrate in the whole progression of AMD. Based on the current result, some specific immune molecular and pathways should be deeply investigated in the further study.

In summary, we performed a comprehensive analysis of the existing high-throughput gene expression datasets in RPE/choroid of AMD. Although there were only a few genes overlapped, probably because of the heterogeneity of AMD, we identified shared pathways and networks made up by the DEGs of these datasets. Our results provided a panorama for better understanding the role of RPE/choroid in the pathogeniesis of AMD. Further, our study might create new avenues of AMD therapeutic strategy by manipulation of these key genes and critical pathways in human RPE/choroid tissues.

## Figures and Tables

**Table 1:** Number of patient samples and differentially expressed genes among GSE29801, GSE50195, and GSE99248.

GEO	No. of samples		No. of changed genes		Method	Reference
	Control	AMD	Up	Down		
GSE29801	31	37	95	55	Transcriptome analysis	Newman et al. [12]
GSE50195	7	9	15	60	Microarray analysis	Whitmore et al. [13]
GSE99248	8	8	67	71	RNA-seq analysis	Kim et al. [14]

**Table 2:** Functional analysis for the DEGs in in RPE/choroid between AMD patients and age-matched healthy controls.

Top diseases and biological functions	*p* value^a^	No. of genes
*Diseases and disorders*		
Cancer	5.96*E*-16-2.41*E*-03	299
Organismal injury and abnormalities	5.96*E*-16-2.41*E*-03	301
Ophthalmic disease	9.88*E*-16-2.26*E*-03	58
Hereditary disorder	2.26*E*-15-2.26*E*-03	75
Neurological disease	2.26*E*-15-2.17*E*-03	126

*Molecular and cellular functions*		
Cell morphology	1.01*E*-12-2.19*E*-03	56
Cellular movement	1.82*E*-10-2.19*E*-03	84
Cellular compromise	6.25*E*-10-1.88*E*-03	27
Cellular development	1.48*E*-09-2.25*E*-03	32
Cellular growth and proliferation	1.48*E*-09-2.25*E*-03	46

*Physiological system development and function*		
Nervous system development and function	7.04*E*-14-1.88*E*-03	88
Embryonic development	1.01*E*-12-2.06*E*-03	55
Organ development	1.01*E*-12-1.86*E*-03	48
Organ morphology	1.01*E*-12-1.69*E*-03	58
Organismal development	1.01*E*-12-2.37*E*-03	87

^a^Range of *p* values indicates a higher level functions that contained multiple lower level functions.

## Data Availability

The GEO data (GSE29801, GSE50195, and GSE99248) supporting this study are available at the NCBI GEO databases (http://www.ncbi.nlm.nih.gov/geo/), which also have been cited at relevant places within the text as references [12–14].

## Supplementary Materials

Supplementary Materials Supplementary Table 1: the summary of clinical information of the normal controls and AMD patients. Supplementary Table 2: the details of clinical information of the normal controls and AMD patients. Supplementary Table 3: the detail information of the 353 DEGs identified in human RPE/choroid tissues. Supplementary Table 4: total significantly enriched biological processes associated with 353 DEGs in human RPE/choroid tissues. Supplementary Table 5: the complete list of identified pathways associated with 353 DEGs in human RPE/choroid tissues. Supplementary Table 6: the *p* values and Log_2_Ratio for each gene in top ten pathways. Supplementary Table 7: 23 predicted networks created by the identified DEGs. Supplementary Table 8: predicted significant activation increased or decreased downstream of biological processes. Supplementary Table 9: immune and inflammation molecular in the upstream regulators. Supplementary Table 10: regulator effect pathways found in RPE/choroid between normal controls and AMD patients.
