# Supplementary material for: Identification of Key Genes and Pathways Associated with Age-Related Macular Degeneration
Source: J Ophthalmol. 2020 Aug 21;2020:2714746. doi: 10.1155/2020/2714746 (PMC7456487; doi:10.1155/2020/2714746)
Supplement: Supplementary Materials — Supplementary Table 1: the summary of clinical information of the normal controls and AMD patients. Supplementary Table 2: the details of clinical information of the normal controls and AMD patients. Supplementary Table 3: the detail information of the 353 DEGs identified in human RPE/choroid tissues. Supplementary Table 4: total significantly enriched biological processes associated with 353 DEGs in human RPE/choroid tissues. Supplementary Table 5: the complete list of identified pathways associated with 353 DEGs in human RPE/choroid tissues. Supplementary Table 6: the p values and Log2Ratio for each gene in top ten pathways. Supplementary Table 7: 23 predicted networks created by the identified DEGs. Supplementary Table 8: predicted significant activation increased or decreased downstream of biological processes. Supplementary Table 9: immune and inflammation molecular in the upstream regulators. Supplementary Table 10: regulator effect pathways found in RPE/choroid between normal controls and AMD patients. [file 2714746.f1.zip › 2714746.f1/S table 1.docx]

Table 2 Summary of clinical information of patients between normal control and AMD patients.

|  | | Control | AMD |
| --- | --- | --- | --- |
| No. of Samples | | 46 | 54 |
| Gender | Male | 27 | 21 |
|  | Female | 19 | 33 |
| Average age | | 81.80±6.57 | 83.80±7.01 |
